# Determining the relationship of kinesiophobia with respiratory functions and functional capacity in ankylosing spondylitis: Erratum

**DOI:** 10.1097/MD.0000000000008226

**Published:** 2017-09-29

In the article, “Determining the relationship of kinesiophobia with respiratory functions and functional capacity in ankylosing spondylitis”,^[1]^ which appeared in Volume 96, Issue 29 of *Medicine*, the affiliation and corresponding author address was missing the name of the university. They should have appeared as:

Department of Physiotherapy and Rehabilitation, Faculty of Health Sciences, Eastern Mediterranean University, Famagusta, Turkey.

Ender AngIn, Department of Physiotherapy and Rehabilitation, Faculty of Health Sciences, Eastern Mediterranean University, North Cyprus via Mersin 10, Famagusta 99450, Turkey (e-mail: ender.angin@emu.edu.tr).
